# Exosomes in the Diseased Brain: First Insights from *In vivo* Studies

**DOI:** 10.3389/fnins.2017.00142

**Published:** 2017-03-23

**Authors:** Efrat Levy

**Affiliations:** ^1^Departments of Psychiatry, Biochemistry and Molecular Pharmacology, New York University Langone Medical CenterNew York, NY, USA; ^2^Center for Dementia Research, Nathan S. Kline Institute for Psychiatric ResearchOrangeburg, NY, USA

**Keywords:** extracellular vesicles, exosomes, brain, *in vivo* studies, neurodegeneration, neuroprotection

## Abstract

Extracellular vesicles (EVs) are nanoscale size vesicles secreted by cells and are important mediators of intercellular communication and genetic exchange. Exosomes, EVs generated in endosomal multivesicular bodies, have been the focus of numerous publications as they have emerged as clinically valuable markers of disease states. Exosomes have been mostly studied from conditioned culture media and body fluids, with the difficulty of isolating exosomes from tissues having delayed their study *in vivo*. The implementation of a method designed to isolate exosomes from tissues, however, has yielded the first insights into characteristics of exosomes in the brain. It has been observed that brain exosomes from murine models of neurodegenerative diseases and human postmortem brains tend to mirror the protein content of the cells of origin, and interestingly, they are enriched with toxic proteins. Whether this enrichment with neurotoxic proteins is beneficial by relieving neurons of accumulated toxic material or detrimental to the brain by propagating pathogenicity throughout the brain remains to be answered. Here is summarized the first group of studies describing exosomes isolated from brain, results that demonstrate that exosomes *in vivo* reflect complex multicellular pathogenic processes in neurodegenerative disorders and the brain's response to injury and damage.

## Introduction

Extracellular vesicles (EVs) are phospholipid bilayer membrane-enclosed vesicles that contain lipids, proteins, and RNA (mRNA and miRNA), secreted by cells into tissue extracellular spaces and biological fluids, and *in vitro*, into cultured conditioned media. *in vitro* studies have shown that cells release subpopulations of EVs with distinct molecular and biological properties (Lai et al., 2016; Willms et al., 2016), with the various species of EVs having different intracellular sites of origin (reviewed in van der Pol et al., 2012; Kowal et al., 2014; Kalra et al., 2016). Plasma membrane-derived microvesicles have a diameter of 100–1000 nm. While these microvesicles are continuously shed from all viable cells, their release from the cell can be triggered under specific pathological conditions (Borroto-Escuela et al., 2015). The EVs species most extensively studied is the exosome, 20–100 nm vesicles formed by the invagination of the membrane of late endosome/multivesicular body (MVB) around cytoplasmic materials (reviewed in Kreimer et al., 2015; van der Pol et al., 2015). Mature exosomes remain inside the lumen of the MVB until they are secreted into the extracellular space when the MVB fuses with the plasma membrane (Stoorvogel et al., 2002). Secreted exosomes are taken-up by target cells, with the exosomal content delivered into the recipient cell (Fevrier and Raposo, 2004). Secreted exosomes were first isolated from the conditioned medium of immature sheep reticulocytes (Johnstone et al., 1987). Since then, *in vitro* studies have shown that exosomes are secreted by various cell types (Simpson et al., 2008), including neurons and astrocytes (Faure et al., 2006). Rat and mouse cortical neurons secrete exosomes in culture that have the typical features of size, density, and saponin sensitivity of exosomes secreted by other cells (Faure et al., 2006). Using proteomic methods, it was also shown that these exosomes resemble exosomes isolated from other non-neuronal cell types, containing typical exosomal markers such as Alix, flotillin, and tumor susceptibility gene-101 (TSG101), but also contain neuron-specific components (Faure et al., 2006).

Exosomes secretion was originally described as a complementary process to the lysosomal and proteosomal degradative pathways for shedding of obsolete membrane and cytosolic proteins (Johnstone et al., 1987). The identification of neuron-specific components associated with exosomes isolated from media conditioned by cultured cells (Faure et al., 2006) suggested that secreted exosomes have roles in cell signaling functions (Record et al., 2011) by shuttling cargo between cells and tissues (Smalheiser, 2007), contributing to the regulation of neurotransmitter receptor levels at the synapse, the production and turnover of myelin membranes proteins, and participating in the progression of neurodegenerative diseases (Gould et al., 2003; Vella et al., 2008; Izquierdo-Useros et al., 2011). A pathogenic function of exosomes has been additionally proposed, suggesting that exosomes can transfer pathogens between cells. One such pathogen that exploits this pathway is the prion protein (PrP), the infectious particle responsible for transmissible neurodegenerative diseases such as Creutzfeldt-Jakob disease of humans and bovine spongiform encephalopathy of cattle (Vella et al., 2008). α-synuclein, involved in the pathogenesis of Parkinson's disease is secreted in a calcium-dependent manner by exosomes (Emmanouilidou et al., 2010). A pathogenic role for exosomes was also proposed for amyloid β (Aβ) (Rajendran et al., 2006) and for phosphorylated tau (Saman et al., 2012), both deposited in the brain of Alzheimer's disease (AD) patients. These data suggest that exosomes may contribute to intercellular membrane exchange and the spread of aggregation-prone proteins throughout the brain. However, exosomes may have a protective function by relieving the cells from toxic accumulation of peptides such as PrP, α-synuclein, as well as Aβ and the amyloid β precursor protein (APP) carboxyl terminal fragments (APP-CTFs) that accumulate intracellularly and disrupt the normal function of the endosomal-lysosomal system to degrade proteins. Thus, I have hypothesized that exosomes secretion plays a pleiotropic role in the brain, both beneficial to the cell by discarding accumulated, toxic material, but also potentially harmful to the brain by contributing to the extracellular build up and transport between cells of toxic material.

Isolating exosomes from *in vitro* sources has provided data on the level and content of exosomes secretion by specific cell types but cannot provide information on the *in vivo* physiological effect on exosomes secretion by multiple cell types under normal, developmental as well as pathological conditions. While exosomes isolated from cerebrospinal fluid (CSF) are enriched in proteins derived from brain, including neuronal and microglia markers, the origin of exosomes in the CSF is not strictly from the brain (reviewed in Vella et al., 2016). Although CSF-derived exosomes can serve as biomarkers for brain disease when changes in specific proteins, lipids, or RNA are detected, there is a need to investigate the changes that occur in the brain environment leading to disease initiation and progression. Moreover, given that in most neurodegenerative disorders the primary changes occur in specific localization within the brain, followed by propagation of pathology into well-defined brain regions, it is likely that the levels of secretion and cargo compositions of exosomes are not homogeneous among various brain regions. Understanding this diversity requires the investigation of exosomes content not only in the brain as a whole but also in distinct brain regions.

Understanding exosomes-based signaling in the brain will likely advance knowledge of neurobiology in the non-affected brain, under normal conditions, in addition to understanding disease-related roles of exosomes. The first protocol to isolate exosomes from brain tissues was developed by Perez-Gonzalez et al. (2012), and a second method has been described, a protein organic solvent precipitation method (Gallart-Palau et al., 2016). Both methods efficiently isolate brain tissue exosomes in amounts sufficient to characterize exosomes by multiple technologies, including “omics” (Gallart-Palau et al., 2016 and Perez-Gonzalez and Levy, personal communication). Given the recent development of such methodology, the number of publications of studies describing brain exosomes is limited. Nevertheless, these studies highlight the unique data that can be obtained by directly isolating and characterizing brain exosomes. This review describes the data obtained from exosomes isolated from brain tissue of patients with neurodegenerative disorders, data that could not be obtained from exosomes isolated from body fluids or conditioned media.

## Method to isolate exosomes from brain tissue

**Figure 1:**
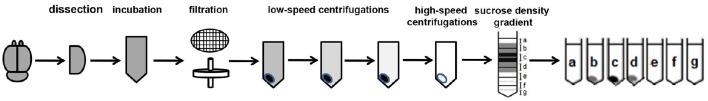
**Brain exosome isolation experimental flow chart**. The steps of the experimental procedure designed to isolate and purify brain exosomes are described.

While exosomes have been successfully isolated from cell culture media and body fluids including CSF, blood, saliva, and urine (Lakkaraju and Rodriguez-Boulan, 2008; Simpson et al., 2008), similar isolation methods for brain exosomes yielded vesicles contaminated with cellular debris, as observed by electron microscopy and Western blot analysis with antibodies to intracellular compartments. We have successfully isolated exosomes-enriched EVs from brain that are not contaminated by cellular debris or intracellular vesicles (Perez-Gonzalez et al., 2012). While this protocol requires rigor and precise performance and we have found that deviation from some of the technique's details can prevent success, several researchers have successfully isolated brain exosomes following our published protocol; their findings are described below. This protocol starts with a gentle dissociation of the tissue to free the brain extracellular space, keeping the cells intact, the same method we have been using for the isolation of intact neurons, glia, smooth muscle cells, and endothelial cells from brain for culture (Jung and Levy, 2005). The brain tissue is not homogenized, but gently disassociated. Unfortunately, in some manuscripts the method has been described as a “homogenization” although the actual technique described involved the gentle dissociation as described in Perez-Gonzalez et al. (2012). After a low-speed centrifugation to discard large debris, the sample is sequentially filtered through 0.4 and 0.2 μm filters to further eliminate remaining debris and vesicles larger than 200 nm, followed by low-speed centrifugations and ultracentrifugations (see Figure 1 and Perez-Gonzalez et al., 2012 for details of our protocol). Electron microscopy of brain EVs demonstrated that this method yields EVs preparations free of debris and subcellular organelles, such as the nucleus, endoplasmic reticulum, mitochondria, and Golgi (Perez-Gonzalez et al., 2012). Western blot analysis demonstrated that the EVs did not contain proteins of intracellular compartments such as endoplasmic reticulum or Golgi, but contained markers of exosomes, indicating the purity of the samples. This procedure yields vesicles that match in shape, size, density, and protein content exosomes previously isolated from body fluids or from the conditioned media of various types of cell cultures (Thery et al., 2006; Simpson et al., 2008). As was previously shown for human urine (Zhou et al., 2006), CSF (Harrington et al., 2009), and plasma (Grant et al., 2011), intact exosomes can be isolated from frozen brain tissues as well. Electron microscopy revealed no difference between exosomes isolated from human brain tissues frozen for several years at −80°C, mouse brains frozen for long periods of time at −80°C, and freshly isolated mouse brains.

Each of the studies that have used this isolation method, described below, employed Western blotting and immuno-EM with exosomes-specific antibodies to identify exosomes within the isolated EVs. These include antibodies to proteins such as the TSG101, required for endosomal sorting complex required for transport (ESCRT) recruitment to endosomal membranes during the process of exosomes generation (de Gassart et al., 2004), multiple rab GTPase, key regulators of intracellular membrane trafficking, including rab27a and rab27b that function in MVB docking at the plasma membrane (Ostrowski et al., 2010), and rab35, which regulates exosomes secretion (Hsu et al., 2010). The endosomal origin of exosomes is supported by the identification of rab5 isoforms (rab5a, rab5b, rab5c), proteins essential for the fusion of early endosomes and regulation of endocytosis, in exosomes (Agola et al., 2011; Pfeffer, 2013). Brain exosomes also contained various tetraspanin proteins, such as CD9, CD63, CD81, abundantly present in the endosomal-lysosomal pathway where they are enriched on the intraluminal vesicles and extensively used as markers of exosomes (Abache et al., 2007; Pols and Klumperman, 2009; Jorgensen et al., 2013).

## Exosomes isolated from brain provide unique data on the role of exosomes in neurodegenerative disorders

Multiple studies have shown that the levels and composition of exosomes are affected by, and contribute to, disease progression (Kreimer et al., 2015) although the mechanisms are poorly understood (Urbanelli et al., 2013). Changes in exosomes generation, secretion, uptake, or clearance, manifested by changes in the number of exosomes in the brain extracellular space, may have either protective or pathogenic effect. Given that exosomes contain amyloidogenic and toxic material, exosomes release can serve a neuroprotective function through the elimination of neurotoxic molecules that cause neuronal endosomal-autophagic-lysosomal abnormalities and functional sequelae, including memory loss. Recent studies have shown that exosomes uptake by microglia is more efficient than by neurons (Fitzner et al., 2011; Yuyama et al., 2012), suggesting that exosomes release may have a neuroprotective function by allowing a neuron to shed deleterious components that are then targeted to and degraded by non-neuronal cells (Joshi et al., 2015). Unfortunately, the elimination of cytotoxic material associated with exosomes by neurons, if not efficiently degraded, may lead to a propagation of toxicity and pathology in the brain, as has been suggested for prion diseases (Vella et al., 2008). The direct isolation of exosomes from the tissue has the advantage of revealing *in vivo* processes in the normal brain as well as changes that occur within the environment of the brain during normal aging or those leading to disease. While regional analyses can be performed, such studies provide limited information on the cell of origin of the exosomes or the mechanisms leading to altered exosomes levels in the brain. To this end, *in vivo* studies often need to be accompanied by *in vitro* studies in which exosomes are isolated from specific cell types, such as neurons, astrocytes, and microglia. This section provides published examples of the unique data obtained from exosomes isolated from brain tissue. While the extent of published studies to date remains limited, these studies and their findings encourage the use of this methodology to further delineate the roles of exosomes secretion, uptake, and signaling in the normal and diseased brain.

### The normal brain

Isolation of exosomes from brain can serve as a confirmation that processes observed in the *in vitro* model also occur *in vivo* in the brain. For example, one study identified a novel mechanism that regulates the activity of the membrane-bound receptor for platelet-derived growth factor A (PDGFRα), crucial for controlling the production of oligodendrocytes for myelination (Pituch et al., 2015). This study tested whether galactosylceramides (sulfatides) contribute to the regulation of oligodendrogenesis by modulating PDGFRα function. It was found that increased sulfatides levels in multipotential neural precursors lead to a reduced production of oligodendrocytes progenitor cells and oligodendrocytes. The activity of PDGFRα depends on its localization to detergent-resistant membrane domains in the plasma membrane and interaction with integrin complexes and therefore, the diminished association of PDGFRα with these domains due to increased sulfatides levels resulted in exacerbated PDGFRα activity. This study identified a novel mechanism that regulates the secretion of PDGFRα in multipotential neural precursor cells, in glial cells, and in the brain cortex via exosomal shedding. While the *in vitro* data has shown that PDGFRα is secreted by multipotential neural precursors and by glial cells via exosomes, the *in vivo* study has shown that PDGFRα is secreted via exosomes during the peak of myelination (Pituch et al., 2015). Exosomal PDGFRα secretion may be a physiologically relevant function of exosomal secretion by which oligodendrocytes progenitor cells rapidly and locally regulate the concentration of the receptor. This can have an impact on the receptor-mediated responses to proliferation, survival and differentiation. Thus, the *in vivo* studies provide further evidence that exosomal regulation of PDGFRα is part of a larger regulatory program involved in myelination.

### Alzheimer's disease

Extensive research has shown that several metabolites of the APP are toxic to neuronal cells. A central pathological feature of AD is the accumulation of Aβ in the brain, and it was suggested that Aβ has an important role in the pathogenesis of neuronal dysfunction in the disease (reviewed in Selkoe, 1991; Hardy and Selkoe, 2002; Stine et al., 2003). Recent *in vitro* and *in vivo* studies show that other APP metabolites, mainly the β-secretase derived APP-CTFs, are neurotoxic and can cause memory loss (Jiang et al., 2010; Deyts et al., 2012; Tamayev and D'Adamio, 2012; Tamayev et al., 2012). Most significantly, it was shown that APP-βCTF cause neuronal endosomal-lysosomal abnormalities and resulting memory loss (Neve et al., 1996; Oster-Granite et al., 1996; Neve and Robakis, 1998; McPhie et al., 2001; Jiang et al., 2010; Deyts et al., 2012; Flammang et al., 2012; Lauritzen et al., 2012, 2016; Oules et al., 2012; Tamayev and D'Adamio, 2012; Tamayev et al., 2012). Exosomal proteins were found to accumulate in the amyloid plaques in the brain of AD patients (Rajendran et al., 2006) and therefore it was proposed that exosomes participate in the pathogenesis of AD. Exosomes isolated from the conditioned medium of neuronal cell cultures contain as cargo the full-length APP, APP metabolites, and key enzymes that cleave both full-length APP and CTFs, and exosomes are one site where APP processing occurs (Rajendran et al., 2006; Vingtdeux et al., 2007; Escrevente et al., 2008; Sharples et al., 2008). The presence of precursors to amyloidogenic proteins and amyloidogenic proteins within exosomes suggest that exosomes can release amyloidogenic material and transmit it between cells, playing a pathogenic role in neurodegenerative diseases.

Isolation of exosomes from mouse brains had shown that murine brain exosomes contain full-length APP and the APP metabolites APP-CTFs and Aβ (Perez-Gonzalez et al., 2012) as was shown previously for exosomes isolated from the media cultured by neuronal cells *in vitro* (Vingtdeux et al., 2007). A novel finding was that exosomes isolated from brains of mice are enriched with APP-CTFs compared to brain homogenates (Perez-Gonzalez et al., 2012). These results imply the existence of a set of mechanisms driving an enrichment of APP-CTFs in exosomes. Additionally brain exosomes were isolated from brains of transgenic mice overexpressing human APP with the K670N/M671L Swedish double mutation (Tg2576; Hsiao et al., 1996). The findings showed that the amount of flAPP and APP-CTFs secreted out of the cell in brain exosomes is proportional to their expression levels in the brain, but that the exosomes are greatly enriched with APP-CTFs (Perez-Gonzalez et al., 2012). Immuno-EM using antibodies directed to cytoplasmic or extracellular epitopes of APP revealed that the exosomes are oriented with the cytoplasmic-side facing inward (Pisitkun et al., 2004). Therefore, cleavage of APP results in capture of APP-CTFs inside exosomes. Thus, the enrichment of APP-CTFs in exosomes isolated from brains results from endosomal APP-CTFs that were captured and carried by exosomes combined with metabolism of flAPP in the exosomes. Contrary to flAPP and APP-CTFs, only a minute fraction of total Aβ (<1%) is associated with exosomes (Rajendran et al., 2006). The orientation of APP in the membrane of exosomes would result in the secretion of exosomal-generated Aβ into the extracellular space, not into the vesicles. Data suggest that Aβ binds to exosomes that possibly act as a seed for plaque formation (reviewed in Dinkins et al., 2016a).

Isolation of exosomes from murine brain also enables direct *in vivo* study of the effect of exosomes release on amyloidogenesis. This was studied in a transgenic mouse expressing five familial mutations in the human APP and presenilin 1 genes (5XFAD). This model primarily produces Aβ42, with amyloid plaques forming as early as at 3 months of age (Oakley et al., 2006). Prevention of exosomes secretion by the inhibition of neutral sphingomyelinase 2 (nSMase2), a key regulatory enzyme generating ceramide from sphingomyelin, with GW4869 in the 5XFAD transgenic mouse reduced total Aβ42 levels and plaque load in the brain (Dinkins et al., 2014). In genetically nSMase2-deficient 5XFAD mice, reduced glial activation, total Aβ42 and plaque burden, and tau phosphorylation, along with an improvement in a fear-conditioned learning task were observed (Dinkins et al., 2016b). These findings are consistent with the idea that APP metabolite loaded exosomes may, under some conditions, contribute to amyloidogenesis. Additionally, the isolation of exosomes from mouse brain facilitated the identification of a difference between females and males, showing that the reduction of total Aβ42 levels and the number of plaques in the brain with a reduction in nSMase2 function was significantly greater in male than female mice (Dinkins et al., 2014), suggesting sex differences in brain exosome secretion or function. This information may provide a mechanism for the higher vulnerability of females to AD pathogenesis: sex differences have been reported in AD, with evidence that women expressing the apolipoprotein E4 allele have a higher risk of being affected by AD than men, regardless of longevity and other disease mortality factors (Payami et al., 1994; Buckwalter et al., 1996; Farrer et al., 1997; Andersen et al., 1999; Bretsky et al., 1999; Lobo et al., 2000; Mortensen and Hogh, 2001; Brookmeyer et al., 2002; Barnes et al., 2003; Baum, 2005; Beydoun et al., 2010; Altmann et al., 2014).

Another major hallmark of AD is the accumulation of pathological tau protein, spreading in well-defined stages between different regions of the brain, potentially via exosomes. Exosomes-associated tau phosphorylated at Thr-181 (AT270), an established phosphorylated tau biomarker for AD, was found in tissue culture media and in human CSF samples, suggesting that exosomes-mediated secretion of phosphorylated tau plays a role in the abnormal processing of tau in early AD (Saman et al., 2012). It was hypothesized that microglia, the primary phagocytes in the brain, facilitate tau protein propagation between neurons by phagocytosing and exocytosing tau, supported by the finding that depleting microglia dramatically suppressed the propagation of tau (Asai et al., 2015). An *in vitro* study had shown that microglia are more efficient at the uptake of tau as compared to neurons and astrocytes. Isolation of exosomes from the brain of a mouse model of tau propagation revealed that vesicles that contain exosomal proteins contain tau and that exosomes isolated from the brain of these mice transduce tau into primary cultured neurons (Asai et al., 2015). Moreover, microglial depletion partially reduced tau content in exosomes, and inhibiting exosomes synthesis significantly reduced tau propagation *in vitro* and *in vivo*. These data suggest that exosomes may play a prominent role in the progression of tauopathy within the brain (Asai et al., 2015).

A study that isolated exosomes from the brain of a tau transgenic mouse that expresses four-repeat tau with the P301L mutation showed that exosomes levels of tau were significantly higher in transgenic mice that have pronounced tau pathology (Polanco et al., 2016). Tau in the vesicles was differentially phosphorylated, although to a lower degree than in the brain cells from which they were derived. Several phospho-epitopes (AT8, AT100, and AT180) thought to be critical for tau pathology were undetected in brain exosomes. Despite this, exosomes derived from transgenic mice were capable of seeding tau aggregation in a threshold-dependent manner, demonstrating that tau-containing exosomes can transmit tau pathology. Moreover, the characteristics of tau in the exosomes and the high seeding threshold identified may explain why tau pathology develops very slowly in neurodegenerative diseases such as AD (Polanco et al., 2016). In another study, the seeding potential of lysates and EVs enriched for exosomes from wild-type and the tau transgenic mouse brains was compared, showing that the transgenic exosomes cause increased tau phosphorylation and soluble oligomer formation in a manner comparable to that of freely available proteins in brain lysates, a prerequisite for the formation of mature protein aggregate (Baker et al., 2016). These *in vivo* studies show that exosomes can propagate tau pathology, can carry tau seeds able to induce tau aggregation in recipient cells, and that such tau is phosphorylated at epitopes found in AD patients.

### Dementia with lewy body and parkinson's disease

α-synuclein aggregation plays a central role in Parkinson's disease pathology. Exosomes released from α-synuclein overexpressing SH-SY5Y cells contain α-synuclein, and these exosomes were capable of efficiently transferring α-synuclein to normal SH-SY5Y cells (Emmanouilidou et al., 2010; Alvarez-Erviti et al., 2011). An *in vitro* study that examined the role of the ESCRT pathway in the propagation of α-synuclein has shown that disruption of the ESCRT transport system resulted in increased exocytosis of α-synuclein (Spencer et al., 2016). The *in vitro* study also showed that the ESCRT protein-charged multivesicular body protein (CHMP2B) affects the release of α-synuclein, and downregulation of CHMP2B affects the intracellular capacity of cells to clear α-synuclein, leading to greater release of α-synuclein into the media. Consistent with these findings, lower levels of CHMP2B were found in the brain of patients diagnosed with dementia with Lewy body as compared with controls, thus generating a roadblock of endocytosed α-synuclein. In order to determine whether α-synuclein is released via exosomes *in vivo*, exosomes were isolated from brain of patients with dementia with Lewy body, revealing that the exosomes contained α-synuclein, while exosomes isolated from control individuals did not (Spencer et al., 2016). These data suggest that neurons compensate for dysfunction of the endosomal-lysosomal pathway that cause accumulation of material such as α-synuclein in MVB by enhanced release of exosomes, potentially increasing cell-to-cell propagation of α-synuclein in neurological disorders with parkinsonism, including Parkinson's disease and dementia with Lewy body.

### Amyotrophic lateral sclerosis and frontotemporal lobar degeneration

Similar to dementia with Lewy body, data on exosomes-associated TDP-43, a pathological hallmark of amyotrophic lateral sclerosis and frontotemporal lobar degeneration, suggest that enhanced exosomes release is beneficial to neuronal survival, in spite of potential increase in disease propagation (Iguchi et al., 2016). *in vitro* investigation of the role of exosomes in the secretion and propagation of TDP-43 aggregates have shown that TDP-43 is secreted by neurons but not by astrocytes or microglia in association with exosomes. Isolation of exosomes from human brains had shown that levels of exosomal TDP-43 full length and C-terminal fragment species are upregulated in human amyotrophic lateral sclerosis brains. When Neuro2a cells were exposed to exosomes from amyotrophic lateral sclerosis brain, but not from control brain, cytoplasmic redistribution of TDP-43 was observed (Iguchi et al., 2016). These data suggest that secreted exosomes contribute to propagation of TDP-43 proteinopathy. However, inhibition of exosomes secretion provoked formation of TDP-43 aggregates in Neuro2a cells and exacerbated TDP-43 neuronal aggregation. Moreover, the disease phenotypes of transgenic mice expressing human TDP-43A315T mutant was exacerbated following *in vivo* inhibition of exosomes secretion. Given that cytoplasmic TDP-43 aggregation occurs in amyotrophic lateral sclerosis and frontotemporal lobar degeneration, these results suggest that exosomes containing pathological TDP-43 play a role in the propagation of TDP-43 proteinopathy. Thus, a therapeutic strategy for amyotrophic lateral sclerosis based on inhibition of exosomes production based on *in vivo* data that suggest that exosomes secretion plays an overall beneficial role in neuronal clearance of pathological TDP-43 would be inadvisable (Iguchi et al., 2016).

### Simian immunodeficiency virus-induced brain disease

Investigation of the role of exosomes in simian immunodeficiency virus (SIV)-induced brain disease was conducted by the isolation and characterization of exosomes from the brains of rhesus macaques. Small RNA sequencing revealed higher miR-21 levels in exosomes from SIV encephalitic brains compared with controls (Yelamanchili et al., 2015a,b). *In situ* hybridization revealed increased miR-21 expression in neurons and macrophage/microglial cells/nodules during SIV induced brain disease. *in vitro* culture of macrophages have shown that miR-21 is released via exosomes and is neurotoxic via activation of TLR7 when compared to exosomes derived from miR-21 knockout mice (Yelamanchili et al., 2015a,b). This study further emphasizes the value of the characterization of exosomal response in the affected brain in combination with an *in vitro* study to further define the mechanism of action.

### Traumatic brain injury

The same exosomes-associated miRNA involved in SIV-induced brain disease was found to have a role in traumatic brain injury (TBI; Harrison et al., 2016). While several experiments have shown the effects of exosomes-associated miRNA on cells in culture, this study investigated how miRNA content of exosomes is altered *in vivo* in the brain. Exosomes were isolated from the brain of a mouse model of TBI (controlled cortical impact) and controls. Profiling of miRNA in the exosomes has shown changes in miRNA species, with miR-21 showing the largest change between conditions. Highlighting the value of isolating exosomes from the brain directly, the miRNA profiles found in this study were not identical to those seen in exosomes isolated from human CSF after TBI described in a previous study (Patz et al., 2013). It remains to be determined whether the difference between the studies is due solely to a difference between CSF and brain exosomes, or due to a difference between TBI in humans and the mouse model, critical to the interpretation of further studies. Study of the mouse brain has shown that the expression of miR-21 in the brain was primarily localized to neurons, not microglia, near the lesion site, while adjacent to these miR-21-expressing neurons were activated microglia. The concurrent elevation of miR-21 in neurons with the increase in miR-21 in exosomes suggests that miR-21 secreted from neurons in association with exosomes is a potential mechanism of cell-to-cell communication resulting in microglia activation as a protective mechanism. Thus, while a neurotoxic function of exosomes-associated miR-21 was shown in the brain of SIV-induced rhesus macaques brain disease (Yelamanchili et al., 2015a,b), a protective function is suggested, but needs to be proven, in a mouse model of TBI (Harrison et al., 2016).

### Schizophrenia and bipolar disorder

In another study, well-preserved exosomal miRNAs were isolated from frozen postmortem prefrontal cortices and submitted to RNA profiling to examine whether they reflect disease-specific changes in psychiatric disorders (Banigan et al., 2013). The expression of certain exosomal miRNA in the brains of patients diagnosed with schizophrenia and bipolar disorder differed from controls, reflecting either disease-specific or common aberrations in these patients. RT-PCR validation confirmed that two miRNAs, miR-497 in schizophrenia brain samples and miR-29c in bipolar disorder brain samples, have significantly higher expression when compared to control samples (Banigan et al., 2013). These results show that brain exosomes characterization has the potential to identify the cellular mechanism of disease pathology, as well as showing the potential of exosomes-derived miRNAs to serve as biomarkers for various disorders.

## Conclusions

Studies of exosomes isolated from brain tissue demonstrate that unique, brain-specific data can be obtained, findings that are not available from exosomes isolated from conditioned media or body fluids. Moreover, they show that exosomes released into the extracellular space of human and mouse brains under disease conditions have different exosomal cargo proteins and miRNAs compared with unaffected controls. However, these *in vivo* studies are not able to identify specific cellular sources of these exosomes as well as cell biological and vesicle trafficking alterations that alter exosomes, data that can be obtained from cultured cells. Given that brain-derived exosomes can reflect disease processes, they may also be used to evaluate the effect of therapeutic interventions on specific markers.

As the field continues to undertake a comprehensive and integrated analysis of brain tissue exosomes, exosomes secreted from the various cell-types found within the brain, and from the CSF, our understanding of exosomes secretion as a cellular rescue pathway and as a potential mechanisms for disease propagation will grow. Additionally, the newly appreciated value of exosomes as biomarkers for disease diagnosis and informers of pathogenic processes within cells will likely lead to greater use of exosomal analyses as translation research and clinical tools.

## Author contributions

EL wrote the review.

## Funding

This work was supported by the National Institutes of Health (AG017617 and AG037693).

### Conflict of interest statement

The author declares that the research was conducted in the absence of any commercial or financial relationships that could be construed as a potential conflict of interest.
